# Targeting CDK9 with selective inhibitors or degraders in tumor therapy: an overview of recent developments

**DOI:** 10.1080/15384047.2023.2219470

**Published:** 2023-06-05

**Authors:** Lanshu Xiao, Yi Liu, Hui Chen, Lisong Shen

**Affiliations:** a Department of Clinical Laboratory, Xinhua Hospital, Shanghai Jiao Tong University School of Medicine, Shanghai, China; b Faculty of Medical Laboratory Science, College of Health Science and Technology, Shanghai Jiao Tong University School of Medicine, Shanghai, China; c Institute of Artificial Intelligence Medicine, Shanghai Academy of Experimental Medicine, Shanghai, China

**Keywords:** CDK9, small molecular inhibitors, PROTAC degraders, tumor, targeted therapy, development

## Abstract

As a catalytic subunit of the positive transcription elongation factor b (P-TEFb), cyclin-dependent kinase 9 (CDK9) has been demonstrated to contribute to carcinogenesis. This review focuses on the development of selective CDK9 inhibitors and proteolysis-targeting chimera (PROTAC) degraders. Twenty selective CDK9 inhibitors and degraders are introduced along with their structures, IC50 values, in vitro and in vivo experiments, mechanisms underlying their inhibitory effects, and combination regimens. NVP-2, MC180295, fadraciclib, KB-0742, LZT-106, and 21e have been developed mainly for treating solid tumors, and most of them work only on certain genotypes of solid tumors. Only VIP152 has been proven to benefit the patients with advanced high-grade lymphoma (HGL) and solid tumors in clinical trials. Continued efforts to explore the molecular mechanisms underlying the inhibitory effects, and to identify suitable tumor genotypes and combination treatment strategies, are crucial to demonstrate the efficacy of selective CDK9 inhibitors and degraders in tumor therapy.

## 1. Introduction

Cancer has surpassed infectious diseases in being the leading cause of death worldwide since the second half of the twentieth century.^1^ In 2020, there were nearly 19.3 million new cancer cases and almost 10.0 million cancer deaths according to the GLOBOCAN 2020 estimates.^2^ Although advances in classical cancer treatment modalities, including surgery, cytotoxic chemotherapy, and radiotherapy, have led to reductions in cancer mortality rates in the past several decades, many challenges still remain to be overcome. Cytotoxic chemotherapy and radiotherapy always lead to severe side effects and cancer cell resistance because they lack specificity.^3^ Given the heterogeneity of cancer, targeted therapies accounting for the array of subtypes, stages, and grades of cancers become imperative.

Cyclin-dependent kinases (CDKs), which are activated by different cyclins at different stages of cell cycle, are characterized as serine/threonine-specific protein kinases in eukaryotes.^4,5^ They play a crucial role in cell proliferation, transcription, differentiation, metabolism, and apoptosis.^5–7^ There are two partially overlapping classes of CDKs-one includes regulators of the cell cycle (CDKs 1, 2, 4, and 6) and the other includes regulators of transcription (CDKs 7–9, 12, and 13) through phosphorylation of the C-terminal domain (CTD) of RNA polymerase II (RNAP II), the largest subunit, and other targets.^5,8,9^

One of the key hallmarks of cancer is dysregulation of cell division, which triggers aberrant cell proliferation. Identifying therapeutic targets that block cell division is a widely used approach in cancer treatment. Therefore, CDKs have long been regarded as promising targets for cancer therapy. The first-generation CDK inhibitors are “pan-CDK” inhibitors, including flavopiridol and seliciclib (roscovitine).^10,11^ In recent years, targeted therapies against CDKs have made considerable progress in the field of tumor therapy, of which CDK4/6 specific inhibitors have been the first to be established as effective. Currently, four CDK inhibitors, targeting CDK4/6, namely palbociclib, ribociclib, abemaciclib, and trilaciclib, have been approved by the FDA. One representative, palbociclib, has been approved for the treatment of advanced estrogen receptor positive (ER+) and human epidermal growth factor receptor 2 negative (HER2-) breast cancer.^12,13^

Transcription-associated CDKs have emerged as important targets in the field of oncology. This family of CDKs plays critical roles in regulating gene expression at multiple levels, including transcription, splicing, intronic polyadenylation, and epigenetic.^14^ Developing specific chemical inhibitors that target the family of CDKs is important. CDK7 inhibitors have exhibited promising potency for tumor treatment, among which CT7001, SY-1365, and SY-5609 have undergone clinical trials (NCT03363893, NCT03134638, and NCT04247126).^15,16^

As a catalytic subunit of P-TEFb, CDK9 plays a critical role in tumorigenesis and progression in a variety of cancers, including hematologic malignancies and solid tumors. An increasing number of selective CDK9 inhibitors and degraders have been developed, some of which were developed mainly for solid tumors. Targeting CDK9 is a promising strategy for cancer therapy. However, only VIP152 has been shown to be clinically beneficial in patients with advanced high-grade lymphoma (HGL) and solid tumors in clinical trials (NCT02635672),^17^ and none of the selective CDK9 inhibitors or degraders have been approved by the FDA. In this review, we introduce the structures, IC50 values, in vitro and in vivo experiments, mechanisms underlying the inhibitory effects, and combination regimens of selective CDK9 inhibitors and degraders. The challenges as well as potentials of targeting CDK9 in tumors using selective inhibitors and degraders are discussed.

## 2. Cdk9

### 2.1 Structure of CDK9

**Figure 1.**
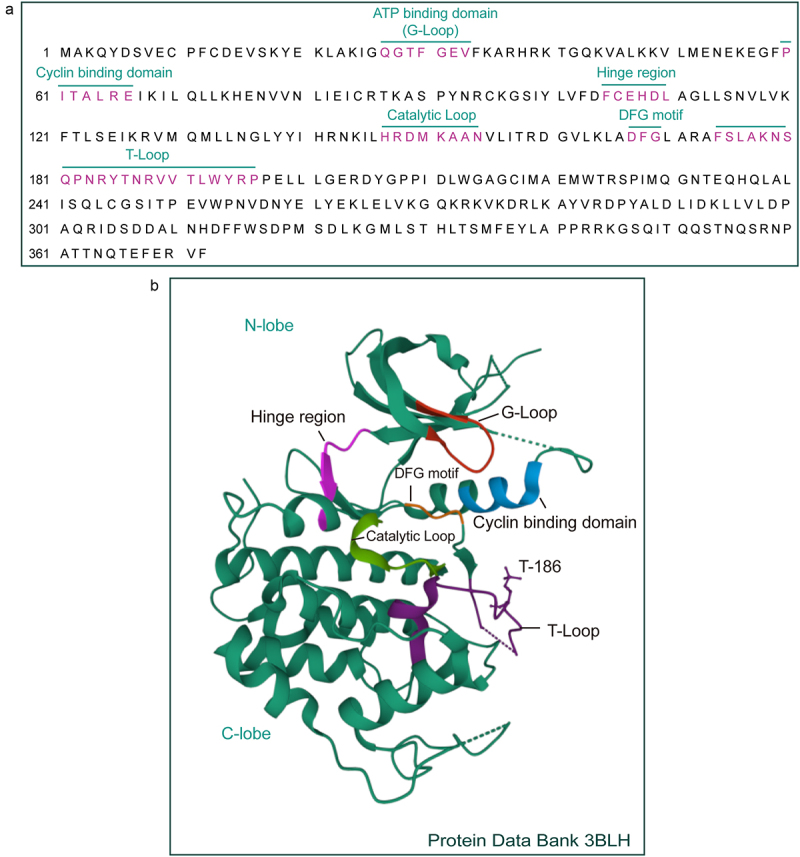
Structure of CDK9.

The CDK9 structure exhibits a typical kinase structure comprising the N-terminal lobe (residues 16–108), which mainly consists of a five β-sheet with one α-helix and the C-terminal lobe (residues 109–330) mainly comprising α-helices. The α-helix on the N-terminal lobe is responsible for the interaction between CDK9 and cyclin T1 (Figure 1). The peptide sequence (PITALRE) in the α-helix is highly conserved across CDKs, and assists cyclin interaction during CDK activation.^18^

### 2.2 Isoforms of CDK9

CDK9 has two isoforms – the major one is 42 kDa and other is 55 kDa.^19^ The 55-kDa isoform (CDK9_55_) has an additional 117 amino acids at the N-terminus, which contains a proline-rich region and a glycine-rich region.^19^ They are encoded by the same gene but are regulated by different promoter regions. The promotor of the CDK9_42_ is characterized as GC-rich sequences,^20^ whereas the CDK9_55_’s transcriptional start, which contains a putative TATA box, is upstream of CDK9_42_’s start point.^19^ But the TATA box was found to be unnecessary for both CDK9_42_ and CDK9_55_ promoter activity.^21^ Additionally, it was found that CDK9_42_ promoter was more active in HeLa cells, activated T cells, and monocytes, which explains the higher protein level in those cells.^21,22^ However, CDK9_55_ is a larger fraction of total CDK9 in inactivated macrophages, mouse lung tissue, and cells modified by viral infection or extracellular signals, suggesting that the relative abundance of the two forms can change.^19^ In contrast to CDK9_42_ protein, CDK9_55_ may be involved in DNA repair through an association with Ku70.^23^ Moreover, CDK9_55_ protein is specifically induced during skeletal muscle regeneration and plays an important part in muscle regeneration process.^24^ Although CDK9_42_ and CDK9_55_ isoforms have identical phosphorylation patterns, they possess different localization. CDK9_42_ is mainly located in the nucleoplasm, whereas CDK9_55_ predominantly accumulates in the nucleus.^21,25^ These results indicate that further efforts are required to discriminate between the functions of CDK9_42_ and CDK9_55_ isoforms.

### 2.3 Roles of CDK9 in transcription

Intricate and efficient regulation of gene expression is the basis of cell growth and development, which mostly occurs during transcription. In the initial step of transcription, multiple factors coordinate with CTD of RNAP II to approach transcription sites, initiate, and elongate transcription.^26^ Transcription elongation has been found to play a substantial role in transcription regulation.^27^ The great majority of RNAP II is found paused at the promoter-proximal regions of most mammalian genes and ready to resume transcription elongation, shortly after RNAP II initiates transcription and synthesizes 20–50 nucleotides of the nascent RNA.^28–30^

**Figure 2.**
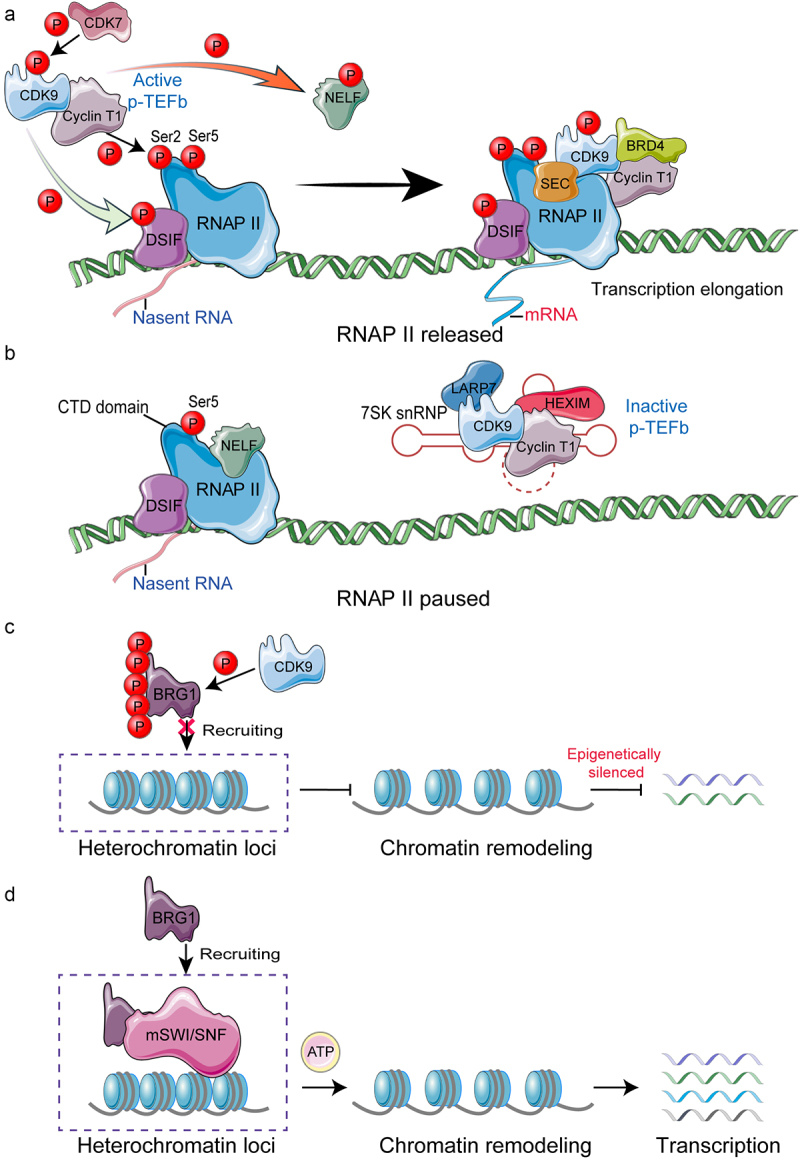
Function of CDK9 in transcription elongation and epigenetic silencing.

The positive transcription elongation factor b (P-TEFb), composed of CDK9 and cyclin T1, can transfer RNAP II from pause to release at the promoter-proximal region by phosphorylating Ser2 of RNAP II’s CTD, the DRB sensitivity inducing factor (DSIF) and negative elongation factor (NELF), then NELF dissociates from RNAP II and transforms DSIF to a state that promoting RNAP II elongation.^31–33^ CDK9, as a catalytic subunit of P-TEFb, contains an ATP site and a T-loop with Thr186 residue that is essential for kinase activity.^34^ To form an activated P-TEFb, CDK9 must be phosphorylated by CDK7 on Thr186.^18^ CDK9 can be activated by combining with cyclin T (T1/T2) or cyclin K (Figure 2a).^35–37^ Remarkably, the phosphorylation of Thr143 and Thr149 in cyclin T1 contributes to its binding to CDK9, which leads to the stability of P-TEFb.^38^

However, more than half of the P-TEFb was reversibly sequestered in an inactive complex (Figure 2b). In this complex, P-TEFb combines with 7SK snRNP, which consists hexamethylene bisacetamide-inducible proteins (HEXIM 1/2) and nuclear RNA 7SK snRNA binding to each other.^39,40^ 7SK snRNA acts as an indispensable scaffold that mediates the interaction between HEXIM 1/2 and P-TEFb.^39^ HEXIM 1/2 contributes to CDK9 inactivation.^4,41^ CDK9 can be inactivated by 7SK snRNP complex, which can reduce the kinase activity of CDK9 by at least 15 times.^18,40,42^ The reversible binding of P-TEFb by 7SK snRNP plays a crucial role in maintaining dynamic balance of gene expression. Recently, it was demonstrated that the dynamic regulation of P-TEFb by 7SK snRNP orchestrated the proper transcriptional response to stress such as cisplatin-induced or ultraviolet light-induced DNA damage.^43,44^

### 2.4 Roles of CDK9 in epigenetic silencing

Brahma-related gene 1 (BRG1), a central component of the SWI/SNF family, is an ATP-dependent helicase that can utilize ATP hydrolysis to regulate chromatin structure.^45^ After the series of five serine residues at the CTD of BRG1 are phosphorylated by CDK9, BRG1 fails to be recruited to the heterochromatin loci (Figure 2c). Inhibition of CDK9 by MC180295 releases BRG1 to regulate chromatin and alter gene expression (Figure 2d).^8^ Hence, CDK9 is essential for maintaining heterochromatin compaction and plays an important role in epigenetic silencing.^8^

## 3. *Roles of* CDK9 in hematological malignancies and solid tumors

**Figure 3.**
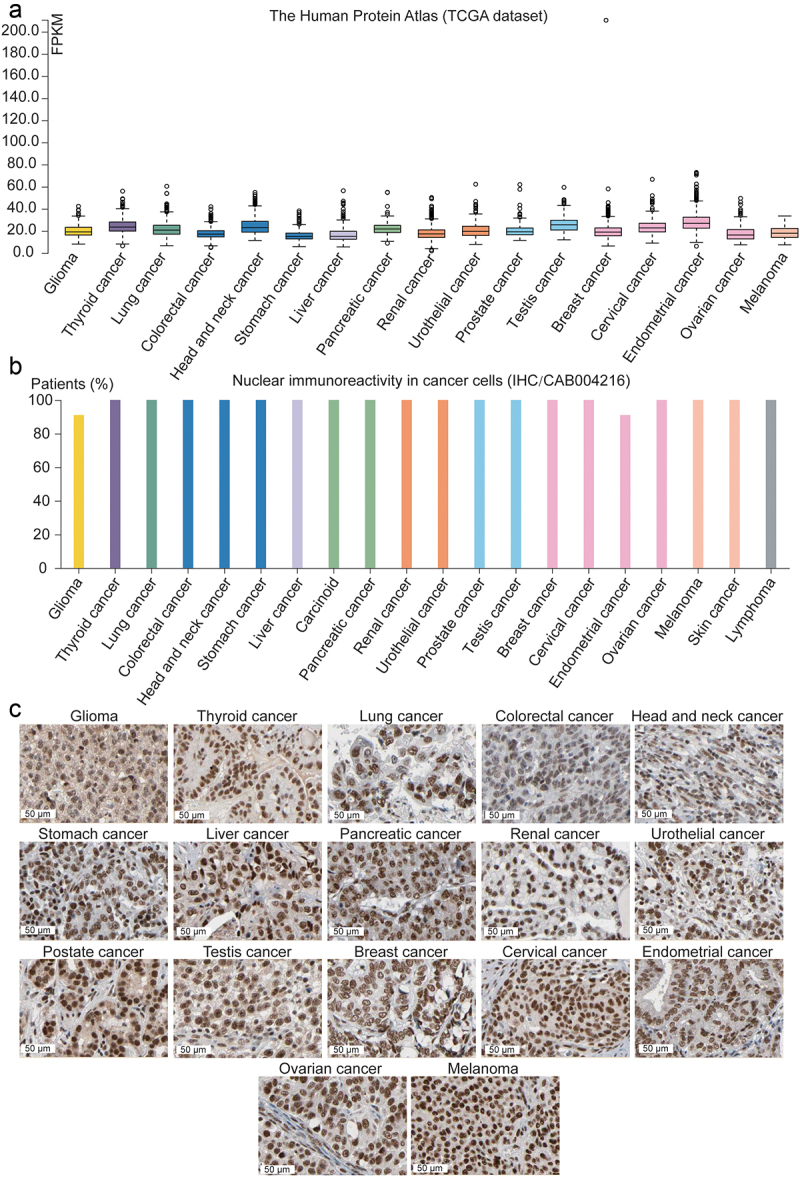
Dysregulation of CDK9 in cancers.

CDK9, which is widely and abnormally expressed in various tumors (Figure 3), has been proven to play an important role in the development and prognosis of a variety of cancers, including hematological malignancies (acute myeloid leukemia [AML], chronic lymphatic leukemia [CLL], B-cell lymphoma, adult T-cell leukemia, multiple myeloma [MM], and NK-cell leukemia),^46–54^ as well as solid tumors (including colorectal cancer [CRC], prostate cancer [PCa], triple-negative breast cancer [TNBC], osteosarcoma, neuroblastoma [NB], medulloblastoma [MB], ovarian cancer, cervical cancer, hepatocellular carcinoma [HCC], esophageal adenocarcinoma [EAC], non-small-cell lung cancer [NSCLC], lung adenocarcinoma, melanoma, and nut midline carcinoma [NMC]).^55–68^

CDK9 has been extensively studied in hematological malignancies. 1) Mixed lineage leukemia (*MLL*) gene on chromosome 11q23 encodes a histone 3 lysine 4 methyltransferase protein (MLL).^69^ *MLL* always undergoes a trans-location mutation fusing with 3’ end of various genes (mainly nuclear transcription factors including AF4, AF6, AF9, AF10, AF17, AF1p, ENL, ELL, and SEPT6) by its 5’ end, accounting for 10% of all acute leukemias.^69,70^ Most of these partner genes are responsible for recruiting P-TEFb and enhancing the expression of *HOX*, *MEIS1*, and *FLT3*, which drive leukemogenesis. Therefore, CDK9 inhibitors (such as dinaciclib and CDKI-73) exert antitumor effects in preclinical models of MLL-rearranged acute leukemia.^71,72^ 2) Myeloid cell leukemia 1 (MCL-1) is upregulated in AML and is essential for AML development and growth.^73,74^ However, short-lived MCL-1 requires P-TEFb to maintain its high expression to promote leukemia cell survival. CDK9 inhibitors reduced MCL-1 protein levels and demonstrated antitumor efficacy in a preclinical AML model.^75^ 3) In AML, CDK9 forms distinct mTOR-like (CTOR) complexes in the cytoplasm and nucleus. In the nucleus, CDK9 binds to RAPTOR and mLST8, forming CTORC1, which promotes the transcription of genes important for leukemogenesis. In the cytoplasm, CDK9 binds to RICTOR, SIN1, and mLST8 to form CTORC2, which controls messenger RNA (mRNA) translation through the phosphorylation of LARP1 and rpS6. Targeting CTORC complexes suppresses the growth of primitive human AML progenitors in vitro and elicits strong antileukemic responses in AML xenografts in vivo.^47^ 4) Other hematological malignancies (including CLL, diffuse large B-cell lymphoma [DLBCL], MM, and NK-cell leukemia) have also been demonstrated to respond to CDK9 inhibitors.^49,50,52–54^

Due to the variety of solid tumors and tumor heterogeneity, CDK9 studies in solid tumors are far fewer than those in hematological malignancies. 1) In estrogen receptor-positive (ERα+) breast tumor, P-TEFb can be recruited by ERα to increase RNAP II Ser2 phosphorylation and overcome transcriptional pausing in intron 1 of the MYB proto-oncogene, leading to tumorigenesis.^76^ However, microRNA-874 can target CDK9 to inhibit proliferation of breast cancer cells and induce apoptosis.^77^ 2) In HCC, CDK9 can mediate transcription elongation of MYC so that the proliferation of MYC-overexpressing HCC is maintained.^63^ CDK9 inhibition by inhibitors or shRNA achieves antitumor effects, which could be predicted at the MYC level.^63^ 3) The phosphorylation of androgen receptor (AR) at serine 81 promotes abnormal growth of PCa cells. CDK9 was reported to be associated with AR and can phosphorylate AR on Ser81 to regulate AR transcription activity and promote PCa growth.^78,79^ 4) In cervical cancer, CDK9 was demonstrated to modulate cell proliferation and apoptosis through upregulating AKT2 and downregulating p53.^62^ 5) In human lung adenocarcinoma, CDK9 was reported to mediate TNF-alpha-induced MMP-9 transcription and tumor invasion and metastasis.^66^ 6) Systematic kinase inhibitor profiling revealed that BRD4-NUT-rearranged NMC cells (HCC2429) are vulnerable to CDK9 inhibitor LDC067.^68^ 7) CDK9 was highly expressed in human osteosarcoma tissues and promoted proliferation of osteosarcoma cells, as demonstrated by the expression of phosphorylated RNAP II Ser2 as well as MCL-1 and BIRC5.^58^ In addition, miR-874 was found to be involved in osteosarcoma progression and metastasis by targeting CDK9.^80^ 8) Other solid tumors (including MB, ovarian cancer, EAC, colon cancer, and melanoma) have also been shown to respond to CDK9 inhibitors.^55,60,61,64,67^

## 4. CDK9 selective inhibitors and degraders and combination regimen

**Figure 4.**
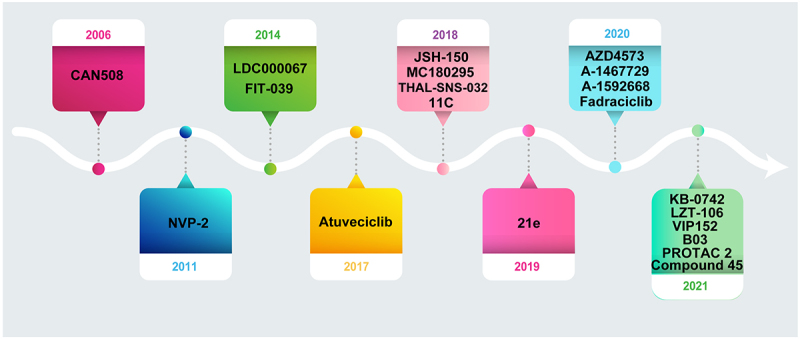
The development of CDK9 selective inhibitors and degraders.

**Figure 5.**
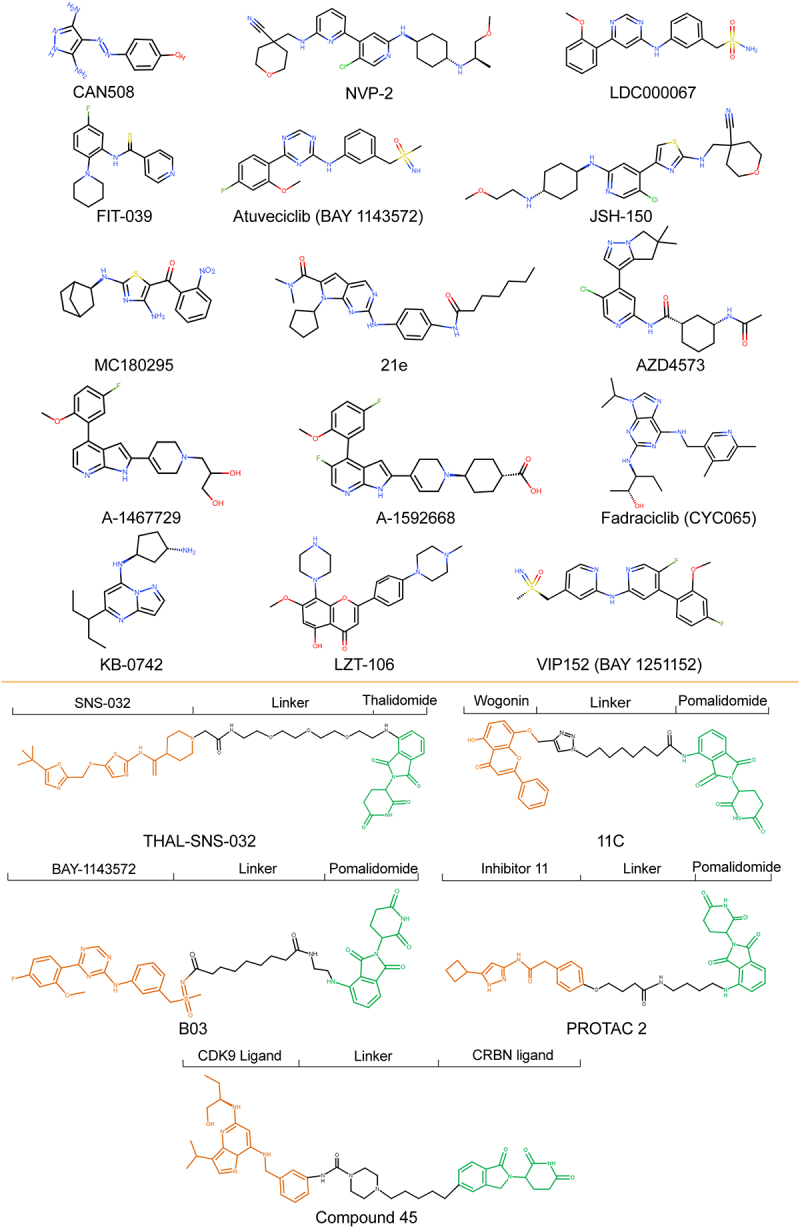
Chemical structures of selective CDK9 inhibitors and degraders.

CDK9 dysregulation leads to uncontrolled oncogene transcription and carcinogenesis; thus, CDK9 is a promising therapeutic target for cancer. The first generation of inhibitors including flavopiridol and seliciclib, often called “pan-CDK” inhibitors, target the ATP-binding site of CDKs, exhibiting suboptimal clinical therapeutic effect.^11,81^ Since the ATP binding region is conserved in the whole CDK family, the first generation of inhibitors is not very specific for CDK9. Some of these inhibitors have been tested in clinical trials, but the adverse effects were serious, and patients had a low rate of complete response.^82–85^ Therefore, selectivity, no off-target activity, and patient tolerance to the inhibitors have received more attention. This review mainly introduces newly emerged CDK9 inhibitors and degraders in recent years, most of which have high potency against CDK9, with a 50% inhibitory concentration (IC50) in the nanomolar (nM) range (Figure 4). Among the degraders, we mainly review those with higher specificity based on proteolysis-targeting chimeras (PROTACs). A summary of the current selective CDK9 inhibitors and degraders, and their potential combination treatment strategies with other drugs are shown in Tables 1 and 2, respectively. The chemical structures of these inhibitors and degraders are shown in Figure 5.

**Table 1.** Summary of current selective CDK9 inhibitors and degraders.

CDK9 inhibitors/degraders	Inhibitory mechanism	In vitro experiments (cell lines)	In vivo experiment (mice tumor model and Clinical Trial)
CAN508^86–88^	Reduce phosphorylation of Ser2/5-RNAP II and decrease MCL-1/VEGF/ICAM-1 in vitro	Reduce proliferation: MCF7 (Breast cancer) OE33, FLO-1 and SKGT4 (EAC) NC160 (59 cell lines panel) Inhibit cell migration and tube formation: HUVEC and HMEC-1 (Primary endothelial cells)	Reduce tumor growth: FLO-1 xenograft mice model (IP, qd for 10 days, 60 mg/kg)
NVP-2^89,90^	Reduce phosphorylation of Ser2-RNAP II	Increase cell death in BRAF^wt^/NRAS^wt^/NF1^wt^ (BNF^wt^) melanoma cell lines (2D and 3D spheroid cultures): CHL (Cutaneous melanoma) OMM2.3, MP41, and MEL202 (Uveal melanoma)	No studies reported yet
LDC000067^91^	Reduce phosphorylation of Ser2-RNAP II, induce p53 and decrease MCL-1	Induce apoptosis: THP1 (Leukemia) Patient-derived blast cells A549 (Lung cancer) HCT116 (CRC) MCF7 (Breast cancer) BRD4-NUT-rearranged cell line (HCC2429) (NMC) Reduce the viability: UCH2 and CH22(Chordoma)	No studies reported yet
FIT-039^92–94^	Reduce phosphorylation of CTD-RNAP II, HPV E6, and E6*I/E7 transcripts and KSHV viral gene	Induce growth inhibition: CaSki (HPV^+^ cervical cancer cells) KSHV^+^ BCBL-1 (PEL)	Reduce tumor growth: HPV16^+^ CaSki subcutaneous tumor xenografts (PO, qd for 3 weeks, 150 or 300 mg/kg) BCBL-1 KSHV^+^ PEL xenograft model (PO, tiw for 45 days, 75, 150, or 300 mg/kg)
Atuveciclib (BAY 1,143,572) ^95–97^	Reduce phosphorylation of Ser2-RNAP II and suppress cFlip, MCL-1 and MDM4	Reduce viability: Primary T-PLL cells FLO-1, SKGT2, SKGT4, OE19, OE33, and ESO26 EAC cells HuH7, HLE, and HepG2 (HCC)	Reduce tumor growth: MOLM-13 xenograft model in mice (PO, QD, 25 mg/kg) MV4–11 human AML model in rats (PO, QD×14, 12 mg/kg) HuH7 xenograft model in mice (PO, five times per week, 6.25, 12.5, or 18.75 mg/kg) Reduced FLO-1 xenograft growth without weight lost Clinical trial: In phase I dose escalation study (NCT02345382) for subjects with acute leukemia (completed) In open label phase I dose escalation study (NCT01938638) for patients with advanced cancer (completed)
JSH-150^98^	Reduce phosphorylation of Ser2-RNAP II and decrease MCL-1 and MYC	Induce growth inhibition: A375 (Melanoma) A431 (Cutaneous SCC) BE (2) M17 (NB) GIST-T1 (GIST) COLO205 (CRC) Ramos, HL-60, MOLM13, MOLM14, OCI-AML-3, SKM-1, U937, MEC-1, MEC-2 (Leukemia cell lines)	In vivo PK/PD evaluation: In mice, Sprague–Dawley rats and beagle dogs (Intravenous injection and oral administration) Reduce tumor growth: MV4–11 xenograft mouse model (PO, QD, 10/20/30 mg/kg/day) without general cytotoxicity
MC180295 ^8,99,100^	Reactivate epigenetically silenced genes by dephosphorylating the SWI/SNF protein BRG1	Reduce proliferation: HCT116 and SW48 (CRC) MCF7 (Breast cancer) DU145 and LnCaP (PCa) KG-1 and HL-60 (Leukemia) YCCEL1 and SNU719 (EBVaGC)	Reduce tumor growth and improve mouse survival: SW48 xenograft NSG mice model (IP, qd for 10 weeks, 20 mg/kg)
21e^101^	Reduce phosphorylation of Ser2/5-RNAP II	Induce growth inhibition: A549, H1299, HCC827 and PC-9 cell (NSCLC exceptionally)	Reduce tumor growth: H1299 xenograft nude mouse model (PO, QD, 20/40/80 mg/kg)
AZD4573^46,97,102,103^	Reduce phosphorylation of Ser2-RNAP II and decrease MCL-1	Induce apoptosis: MCF7 (Breast cancer) MV4–11 (Leukemia) MOLP-8 (MM) OCI-LY10 (Lymphoma), NCI-H2122 (Lung cancer) 211 other cell lines Nine AML PDX models (IV) T-cell lymphoma PDX model HuH7, HLE, and HepG2 (HCC)	Reduce tumor growth: MV4–11 subcutaneous tumor xenografts (IP, BID q2h, on a 2-day on/5-day off schedule, 5 or 15 mg/kg) HuH7 xenograft model in mice (IP, biw, 10, 15, or 20 mg/kg) Clinical trial: In phase I clinical study (NCT03263637) as monotherapy for relapsed/refractory hematological malignancies (completed). In phase I clinical study (NCT04630756) as monotherapy for advanced blood cancer (recruiting). In phase II clinical study (NCT05140382) as monotherapy or in combinations with anticancer agents for r/r PTCL or r/r cHL (recruiting)
A-1467729, A-1592668^104^	Reducing phosphorylation of Ser2-RNAP II	Reduce cell viability and induce apoptosis: H929 and MV4–11 (Leukemia)	Reduce tumor growth: MV4–11 subcutaneous tumor xenografts (PO, biw for 3 weeks, 5 mg/kg)
Fadraciclib (CYC065)^105^	Reduce phosphorylation of Ser2-RNAP II and decrease MCL-1 and RB pThr821	Induce apoptosis: Cal51 and MDA-MB-468 (TNBC) HER2-amplified HCC1954 (Breast cancer) Growth inhibition and cell death: Most AML cell lines Kelly, BE (2)C, IMR32, L-AN-5, SH-EP-MYCN (MYCN elevated expression cell) Primary CLL cells	Induce significant tumor growth inhibition and increase overall survival: Mice carrying MYCN-amplified Kelly NB tumor xenografts Clinical trial: In phase I/II clinical study (NCT05168904) as monotherapy for leukemia or MDS (recruiting) In phase I/II clinical study (NCT04983810) as monotherapy for advanced solid tumors (endometrial or ovarian cancer, biliary tract cancer, HCC, breast cancer) and lymphoma (recruiting)
KB-0742^56^	Reduce phosphorylation of Ser2/5/7-RNAP II and decrease AR and phospho-AR levels (Ser81)	Induce apoptosis and growth inhibition: 22Rv1 (Pca) MV4–11 (Leukemia)	Reduce tumor growth: 22Rv1-driven CDX models (PO, QD, 3, 10, and 30 mg/kg, over 21 days) MV4–11-driven human AML CDX model (PO, QD, 25 mg/kg) (PO, 3 days on/4 days off, 15, 30, and 60 mg/kg) Clinical trial: In phase I dose escalation and cohort expansion study (NCT04718675) for participants with relapsed or refractory solid tumors (small cell lung cancer (SCLC) and soft tissue sarcomas) or Non-Hodgkin Lymphoma (recruiting)
LZT-106^55^	Reduce phosphorylation of Ser2-RNAP II, decrease MCL-1 by regulating GSK-3β signaling	Induce of apoptosis: RKO, HCT116, and HT-29 (CRC)	Reduced tumor growth: RKO xenograft mice (IV, QOD for 14 days, 5/10/20 mg/kg) (no obviously toxic effects observed in all treatment groups)
VIP152 (BAY 1,251,152)^17,106^	Decrease MYC, MCL-1 and PCNA	Induce growth inhibition: A2780 (Ovarian cancer) MOLM-13 (Leukemia)	Reduce tumor growth: The MOLM-13 xenograft model in rats (IV, Q3D (3 mg/kg), Q5D (3.75 mg/kg), or QW (4.5 mg/kg)) with excellent tolerability The MOLM-13 xenograft model in mice (IV, QW, 12.5 mg/kg) with no significant body weight reduction MV4–11 xenograft model in rats (IV, Q7D × 3, 4.5 mg/kg) with complete remission in all animals with no measurable tumor burden for an additional 30 days Eµ-MTCP1 CLL xenograft model in mice (IV, Q7D for 14 weeks, 12.5 mg/kg) with no significant body weight reduction Clinical trial: In phase I study (NCT04978779) for subjects with relapsed/refractory chronic lymphocytic leukemia or richter syndrome (recruiting) In phase I dose escalation study (NCT02635672) for patients with advanced cancer (13 kinds) (recruiting) In phase I trial (NCT05371054) to test VIP152 in combination with venetoclax, and prednisone in relapsed/refractory lymphoid malignancies (not yet recruiting)
THAL-SNS-032^107,108^	Degrade CDK9	Induce caspase3-dependent apoptosis: MCF7, T47D and BT474 (Breast cancer cell lines with ER+)	Induce on-target off-tumor effect (Severe gastrointestinal disorder): BT474 xenograft mice model (IP, qw for 1 month, 10 mg/kg; IP, biw for 1 month, 5 mg/kg; IP, tiw for 1 month, 2.5 mg/kg)
11C^136^	Degrade CDK9	Reduce proliferation and induce apoptosis: MCF7 (Breast cancer)	No studies reported yet
B03^109^	Degrade CDK9, reduce phosphorylation of Ser2/5-RNAP II and decrease MCL-1	Reduce proliferation: MV4–11, MOLM13 (AML) HCT-116 (CRC), B16 (Melanoma cell) A549 (Lung cancer) HepG2 (HCC) MCF7 (Breast cancer) Induce apoptosis: MV4–11 (AML)	Degrade CDK9 in vivo: MV4–11 xenograft mice model (IV, 3 h/6 h, 20 mg/mL)
PROTAC 2^110^	Degrade CDK9	Sensitize pancreatic cancer cell to BCL2 inhibitors: MiaPaCa2 (Pancreatic cancer)	No studies reported yet
Compound 45^57^	Degrade CDK9 and reduce MYC	Reduce proliferation: MDA-MB-468, MDA-MB-231 and BT-549 (TNBC)	Inhibit tumor growth: MDA-MB-231 xenografts mice model (IP, qd for 7 days, 2.5 or 5.0 mg/kg) with slight body weight loss

**Table 2.** Combined use of CDK9 inhibitors or degraders with other drugs.

Combined application of CDK9 inhibitor or degrader with other drugs		Experimental models (In vitro and/or in vivo)	Synergy Effects
LDC000067	Cisplatin (First generation platinum anticancer drugs)^60^	HD-MB03 and ONS-76 MB cells	Reduce MB cells growth
	JQ1 (BRD4 inhibitor)^60,68^	HD-MB03, ONS-76, D283, and DAOY MB cells; HCC2429 NMC cells	Reduce MB cell growth, migration, and gene expression
Atuveciclib (BAY 1,143,572)	Venetoclax (BCL2 inhibitor)^111^	Primary T-PLL cells	Achieve a higher antileukemic effect
	izTRAIL (Recombinant tumor necrosis factor-related apoptosis-inducing ligand)^112^	PDAC cells and PDAC PDX cell lines	Reduce PDAC cells growth by inducing apoptosis and cell-cycle arrest
	Nutlin-3a (Inhibitor of MDM2- p53 interaction)^96^	A375 and MEL-JUSO melanoma cells	Kill A375 and MEL-JUSO cell
	5-fluorouracil^64^	OE33 cells, FLO-1, and SKGT4 EAC cells FLO-1 and ESO-26 murine xenografts	Reduce EAC cells proliferation and induce apoptosis
AZD4573	Venetoclax (BCL2 inhibitor)^46^	SU-DHL-4 and OCI-AML3 cells (Leukemia) SU-DHL-4 and OCI-AML3 subcutaneous xenograft models	Produce highly durable regressions in 100% of treated animals, with all eight mice remaining tumor-free out to day 63. And minimal body weight loss was observed.
A-1592668	Venetoclax (BCL2 inhibitor)^104^	SU-DHL-4, OCI-Ly1 199R, and SC-1 199R cells, AML cell lines including SKM-1 (Leukemia) SU-DHL-4 xenograft model DLBCL and FL lymphoma patient samples ex vivo SKM-1 xenograft model and AML PDX models	Produce greater tumor growth inhibition
Fadraciclib (CYC065)	ABT-263/ABT-737 or venetoclax (BCL2 inhibitor)^105^	HEL, THP-1, and PL21 (Some AML cell lines not dependent on the depletion of MCL-1)	Induce widespread apoptosis efficiently
	Venetoclax (BCL2 inhibitor)^113^	Primary CLL cells	Induce more profound CLL cell death, especially in samples with 17p deletion
	Temozolomide (DNA-damaging agent)^114^	TH-MYCN murine model of NB	Achieve tumor eradication and remarkable extension of overall survival
LZT-106	Venetoclax (BCL2 inhibitor)^55^	RKO CRC cell line	Induce apoptosis of RKO cells
PROTAC 2	Venetoclax (BCL2 inhibitor)^110^	MiaPaCa2 pancreatic cancer cell line	Reduce MiaPaCa2 pancreatic cancer cell growth

### 4.1 Selective CDK9 inhibitors

The selective CDK9 inhibitors reviewed below inhibit the activity of CDK9, but do not affect the expression of CDK9. Most inhibitors inhibited CDK9 by reversibly binding to the ATP pocket, except for JSH-150, A-1467729, and A-1592668. JSH-150 formed hydrogen bonds with CDK9 at the Cys106, Asp109, and Asp167 sites instead of the ATP-binding pocket. However, the inhibitory mechanisms of A-1467729 and A-1592668 have not yet been described.

#### Can508

CAN508 is a 4-arylazo-3,5-diamino-1 H-pyrazole CDK inhibitory compound designed by Krysˇtof et al.^86^ It selectively inhibits CDK9 kinase by reversibly binding to the ATP pocket in vitro. CAN508 decreases the phosphorylation of Ser2 and Ser5 at the CTD of RNAP II and reduced the MCL-1 protein.^86–88^

CAN508 has been shown to exhibit anti-proliferative activity toward tumor cell lines (MCF7, OE33, FLO-1, and SKGT4).^86^ In addition, CAN508 was demonstrated to have antiangiogenic activity in cell migration and tube formation toward primary endothelial cells (HMEC-1 and HUVEC) by reducing VEGF and ICAM production.^87^ In xenograft models (FLO-1), CAN508 caused reduction of tumor growth without significant signs of toxicity as monitored by body weights.^88^

#### NVP-2

NVP-2, a highly selective aminopyrimidine-derived ATP-competitive CDK9 inhibitor, was designed by Novartis.^89^ It has sub-nanomolar potency for CDK9 (IC50 < 0.514 nM).^107^ The main effect of NVP-2 on CDK9 is to inhibit kinase activity and decrease the phosphorylation of Ser2 at CTD of RNAP II.^90,107^

NVP-2 demonstrated greater suppression of BRAF^wt^/NRAS^wt^/NF1^wt^ (BNF^wt^) cutaneous and uveal melanomas than mutant melanomas at 500 nM. Because BNF^wt^ melanoma cells are more dependent on the E2F transcriptional network than BNF^mut^ cells, they are more vulnerable to NVP-2. Human melanoma tumor data from the Cancer Genome Atlas (TCGA) also support the idea that E1F1 and E2F2 are potential oncogenes in BNF^wt^ tumors and are directly linked to CDK9. NVP-2 is an effective inhibitor of therapeutically orphaned BRAF/NRAS/NF1 triple wild-type melanoma.^90^

#### Ldc000067

LDC000067 (abbreviated as LDC067), a compound based on a 2,4-aminopyrimidine scaffold with specific ATP-competitive power, was highly selective against CDK9 (IC50 = 44 nM). Its selectivity for CDK9 over other CDKs ranges from 55-fold (vs. CDK2) to 230-fold (vs. CDK6 and CDK7). It is characterized by high stability, good tolerability, and low cytotoxicity.^91^ LDC067 promotes the apoptosis of several cell lines, especially cells of leukemia origin through reducing RNAP II Ser2 phosphorylation and inducing p53 activation. In addition, LDC067 forces RNAP II to pause and accumulate at promoter-proximal sites, limiting CDK9 activity; thus, RNA synthesis of cellular genes is downregulated.^91^

Inhibition of CDK9 by LDC067 suppressed MB cell growth and enhanced susceptibility of MB cells to cisplatin. Moreover, LDC067 in combination with BRD4 inhibitor decreased MB cell growth, delayed cell migration, and attenuated RNAP II Ser2 occupancy to CCND1 and BCL2 gene promoters.^60^ CDK9 inhibition by LDC067 reduces cell growth of BRD4-NUT-rearranged NMC cells and induces apoptosis.^68^ Additionally, it decreased cell growth and colony formation of chordoma cells.^115^ In addition, overlapping effects of LDC067 and JQ1 on MYC expression and growth inhibition were observed.^68^ The synergistic effect of LDC067 and BRD4 inhibitor is due to BRD4 being responsible for recruiting P-TEFb complex and contributing to transcriptional elongation.

LDC067 plays an important role in suppressing RANKL-induced osteoclast genesis, delaying subchondral osteolysis, and ameliorating LPS-induced osteolysis by downregulating the expression of osteoclast-related marker genes, such as cathepsin K (CTSK).^116^

#### FIT-039

FIT-039 suppresses kinase activity of CDK9/cyclin T1 (IC50 = 5.8 μM) in ATP-competitive manner in vitro, and it does not inhibit other CDKs including CDK2, CDK4, CDK5, CDK6, and CDK7.^92^ FIT-039 inhibits replication of the HSV-1 genome in a dose-dependent manner, which may be attributed to the inhibition of the viral transcription via the suppression of the CTD phosphorylation of RNAP II. FIT-039 was demonstrated to suppress the replication of a broad spectrum of DNA viruses.^92^

FIT-039 suppressed both HPV E6 and E6*I (E6-spliced form)/E7 transcripts in a dose-dependent manner, and p53 and pRb tumor suppressors targeted by HPV E6 and E7 were restored after FIT-039 treatment.^93^ Additionally, FIT-039 treatment showed growth-suppressive effects comparable to those of E6 and E7 knockdown in CaSki cells (HPV^+^ cervical cancer cells).^93^ A significant retardation of HPV16^+^ CaSki xenograft tumor growth was observed in mice treated with 300 mg/kg BW FIT-039.^93^

Another report found that Kaposi’s sarcoma-associated herpesvirus (KSHV) viral gene expressions were dependent on CDK9 and was susceptible to FIT-039 treatment.^94^ KSHV^+^ cells were sensitive to FIT-039 treatment showing growth suppression. FIT-039 administration suppressed the proliferation of KSHV^+^ PEL (primary effusion lymphoma) in a xenograft model.^94^

#### Atuveciclib (BAY 1,143,572)

Atuveciclib (BAY 1,143,572)^117^, the first tolerable oral ATP-competitive CDK9 inhibitor, exhibited highly selective CDK9 inhibition (IC50 = 13 nM) in vitro under low ATP conditions (ATP concentration = 10 μM) and promising anti-proliferative potency toward several human cancer cell lines. Its effect against CDK9 was more than 50-fold greater than that against other CDKs. However, its inhibition effect on CDK9 significantly drops (IC50 = 380 nM) under high ATP conditions (ATP concentration = 2 mM), which is the appropriate cellular ATP condition in human, and limited therapeutic capacity was found in a phase I clinical trial.^95^ Moreover, because of targeting MCL-1 in white blood cells, atuveciclib caused neutropenia as the main adverse event in patients with advanced cancer.^95^

Atuveciclib exerted strong antileukemic activity by inducing apoptosis in T-cell prolymphocytic leukemia (T-PLL) cells. Additionally, the combination of atuveciclib and venetoclax resulted in a greater antileukemic effect than that with monotherapy with each.^111^ Atuveciclib is synergized with TRAIL to inhibit the viability of pancreatic ductal adenocarcinoma (PDAC) cancer cells through the concomitant suppression of cFlip and MCL-1.^112^ Atuveciclib downregulated MDM4 and upregulated p53 activity induced by nutlin-3a (an inhibitor of MDM2-p53 interaction) and synergized with nutlin-3a in killing A375 melanoma cells.^96^ Both MDM2 and MDM4 are important negative regulators of p53 protein, and CDK9 activity was identified as critical for maintaining MDM4 levels, which can explain synergistic effect of atuveciclib and nutlin-3a. Atuveciclib inhibits the binding of HIF-1α to MCL-1 promoter and reduces MCL-1 expression; therefore, plays an antitumorigenic role in EAC.^64^ The combination of atuveciclib and 5-fluorouracil enhanced the downregulation of the MCL-1 protein in vitro, and they synergized in in vitro models as well as murine xenografts of EAC.^64^ Atuveciclib is the first highly selective CDK9 inhibitor to enter clinical trials (NCT02345382 and NCT01938638) for the treatment of cancer.^117^

#### JSH-150

JSH-150 is a highly selective CDK9 inhibitor (IC50 = 1 nM), which achieves approximately 300–10000-fold selectivity against CDK9 over other CDK family members. Unlike most other CDK9 inhibitors that interact with the ATP-binding pocket, JSH-150 forms two hydrogen bonds with Cys106 in the hinge-binding region, a hydrogen bond with Asp109, a hydrogen bond with Asp167 in the activation loop and a hydrogen bond with the Thr29 in the P-loop. It dose-dependently inhibited the phosphorylation of RNAP II CTD (Ser 2), and downregulated MCL-1 and MYC to induce apoptosis and arrest the cell cycle at the G0/G1 phase in leukemia cells.

JSH-150 displayed potent anti-proliferative effects against melanoma, cutaneous squamous cell carcinoma, NB, gastrointestinal stromal tumors (GISTs), CRC, and leukemia cell lines. In an MV4–11 cell-inoculated xenograft mouse model, a 10 mg/kg dose of JSH-150 almost completely suppressed tumor progression. During in vivo PK/PD evaluations of JSH-150, administration to Sprague – Dawley rats and beagle dogs through intravenous injection, and oral administration, indicated that JSH-150 is suitable for oral administration.^98^

#### Mc180295

MC180295 is a novel selective ATP-competitive CDK9 inhibitor discovered by Zhang et al, which has an aminothiazole core structure.^8^ The norbornyl group from MC180295 requires that the C-terminus of the hinge region adopts a slightly lower conformation, which is observed in many crystal structures of CDK9. This conformational flexibility distinguishes CDK9 from other CDKs; it can fit in the norbornyl group of MC180295, whereas other CDKs cannot.^8^ More importantly, Zhang et al. discovered CDK9 was also essential for maintaining gene silencing at heterochromatic loci. CDK9 inhibition using MC180295 reactivates epigenetically silenced genes in cancer by dephosphorylating the SWI/SNF protein BRG1, leading to the restoration of tumor suppressor gene expression, cell differentiation, and activation of endogenous retroviral genes.^8^

MC180295 exerts antitumor effects both in vitro and in vivo. It was more effective at reducing the proliferation of cancer cells (including HCT116, SW48, MCF7, DU145, LnCaP, KG-1, and HL-60) than in the normal lung fibroblast cell line (IMR90). MC180295 treatment resulted in an increase in the sub-G1 subpopulation.^8^ MC180295 inhibits the growth of EBVaGC cells by suppressing DNA repair and the cell cycle.^99^ After treating with MC180295, the genes belonging to “role of BRCA1 in DNA damage response” and “cell cycle control chromosomal replication” were significantly downregulated in both YCCEL1 and SNU719 cells.^99^ In addition, MC180295 significantly induced the expression of the differentiation marker CD11b in HL60 cell line. In CRC mouse models established by injecting SW48 into NSG mice, MC180295 slowed tumor growth and improved mouse survival without overt toxicity, as measured by body weight.^8^ Additionally, MC180295 is harmless to human T lymphocytes, and did not affect the ratio of CD4^+^ and CD8^+^ T cells in vivo. These data indicate that MC180295 is a promising antitumor inhibitor.^8^ However, there is evidence that MC180295 is less effective than AZD4573 in melanoma cell lines.^100^

#### 21e

21e is a promising and selective oral CDK9 inhibitor (IC50 = 11 nM), which was designed by Wang et al. based on the structure of ribociclib (inhibitor of CDK4/CDK6).^118^ Compound 21e binds to the ATP-binding sites of CDK9 in similar orientation to that of ribociclib.^65^ The result of 21e being investigated against a series of 381 kinases by the Eurofins kinases profiling at a single concentration of 1 mM showed that 21e effectively inhibited the activities of CDK9/cyclin T over other CDKs.^65^ 21e inhibits phosphorylation of RNAP II CTD (Ser 2 and Ser 5) dramatically in A549 and H1299 NSCLC cells.^65^

21e significantly inhibited the colony-forming ability of NSCLC cells and induced cell cycle arrest in the G2/M phase by inhibiting the phosphorylation of RNAP II CTD (Ser 2/5). 21e displayed exceptional potency against NSCLC (IC50 < 0.5 μM) and against other cancer cell lines (breast cancer, HCC, cervical cancer, leukemia, and lymphoma). Additionally, 21e efficiently inhibited the stemness properties of NSCLC cells.^65^ In vivo, the H1299 xenograft nude mouse model was significantly inhibited by 21e in a dose-dependent manner without obvious toxicity.^65^

#### Azd4573

Cidado et al. identified a selective and potent ATP-competitive CDK9 inhibitor AZD4573 with structure-based drug discovery method (IC50 at K_M_ and high ATP concentrations are <0.003 μmol/L and <0.004 μmol/L, respectively).^46^ They found that AZD4573 exhibits more than 25-fold selectivity for CDK9 over other CDKs upon short-term treatment of MCF7 cells, which have a frameshift mutation in CASP3 that prevents apoptosis induction and cell death.

Transient exposure to AZD4573-induced apoptosis in MV4–11 cells (as well as MOLP-8, OCI-LY10, and NCI-H2122 cell lines, although some cancers rely on survival factors other than MCL-1) mainly by reducing the phosphorylation of pSer2-RNAP II, which led to a decrease in MCL-1.^46,102^ Therefore, AZD4573 has a potential antitumor effect on MCL-1-dependent hematological malignant tumors. Greater activity of AZD4573 was observed across hematological cancers compared with solid tumor cell lines, although a subset of solid tumor cell lines showed sensitivity (216 cell lines were included in the experiment).^46^ Intermittent dosing of AZD4573-induced regression of MV4–11 tumor xenografts, resulting in regressions for all mice that were sustained for more than 125 days with 15 mg/kg dose.^46^ Moreover, AZD4573 demonstrates antitumor activity in disseminated leukemia and lymphoma patient-derived xenograft (PDX) models.^46^ Five of the nine AML PDX models exhibited >50% reduction of leukemic blasts in the bone marrow after AZD4573 treatment. In the angioimmunoblastic T-cell lymphoma PDX model (DFTL-78024), AZD4573 treatment led to significantly more CD45^+^ tumor cells undergoing apoptosis. AZD4573 rapidly downregulated pSer2-RNAP II, Bfl-1, and MCL-1, increased caspase-3 cleavage, and induced apoptosis in BH3-mimetic – resistant lymphoma cell lines in vitro. It induced in vivo tumor regressions in DLBCL xenografts (OCILY10 and TMD8) and PDX models expressing Bfl-1.^103^ A combination of AZD4573 and c-MYC inhibitor 10,058-F4 can effectively suppress araC-resistant AML.^119^

Venetoclax has been approved for treatment of adult patients with CLL; however, acquired resistance to venetoclax is beginning to emerge, and one mechanism identified preclinically is compensation by increased levels of MCL-1.^120^ In vitro and in vivo models with both SU-DHL-4 and OCI-AML3 showed that combination of AZD4573 with venetoclax effected highly durable regressions in 100% of the treated animals, with all eight mice remaining tumor-free till day 63.^46^ Monotherapy treatment with venetoclax was minimally efficacious, and single-agent AZD4573 again exhibited extensive tumor growth inhibition, but not regressions. Notably, minimal body weight loss was observed, indicating that the combination regimen was tolerated.

#### A-1467729 and A-1592668

A-1467729 was identified as a potent CDK9 inhibitor (IC50 = 1.2 nM) with > 1000-fold selectivity for CDK9 over CDK1, 2, 7, and 8. After treatment, pSer2-RNAP II decreases at the cellular level, with a potency of 26.7 nM. A-1592668 was obtained by modifying the right portion of A-1467729. A-1592668 retained its potency against CDK9 (IC50 = 2.6 nM) with more than 500-fold selectivity over CDK7 and CDK8. They have similar affinities toward CDK9, but A-1592668 has lower clearance, so it can be used to assess CDK9 inhibition effects in vivo.^104^

A-1592668 showed strong pharmacodynamic marker movement and cell killing in MCL-1 dependent cell lines. Treatment with A-1592668 in H929 or MV4–11 cells induced the loss of MCL-1 expression, correlating with a decrease in pSer2-RNAP II, accompanied by apoptosis, as indicated by caspase-3 processing, PARP cleavage, and phosphatidylserine externalization. In addition, A-1592668 induced regression of MV4–11 tumors in vivo, which was maintained for up to 35 days post-treatment without dramatically affecting weight loss. Similar effects have been observed in murine #4242 E*µ-myc* lymphoma cells, B-cell lymphomas deficient in p53 (#3391), and #4242 tumors transplanted into immunocompetent mice.^104^

MCL-1 expression renders a critical resistance factor limiting venetoclax activity in many hematologic tumor types including lymphoma,^121–123^ whereas BCL2 overexpression in Eµ-myc tumors inhibited A-1592668 activity in vitro.^104^ A-1592668 is synergized with venetoclax to inhibit the viability of SU-DHL-4, OCI-Ly1 199 R, and SC-1 199 R cells, and this combination led to greater tumor growth inhibition than did each agent independently in SU-DHL-4 xenograft model.^104^ Similar inhibitory effects were observed in primary DLBCL and follicular lymphoma (FL) samples ex vivo, AML cell lines (including SKM-1), SKM-1 AML xenograft model, and AML PDX models (PDX 4,095,636 and PDX 3,899,936) with this combination.^104^

#### Fadraciclib (CYC065)

Fadraciclib was obtained by further optimizing the aminopurine scaffold of seliciclib (an ATP-competitive inhibitor of CDK2, CDK7, and CDK9). Compared with seliciclib (IC50 = 13.3 μM), fadraciclib is more selective for CDK2 (IC50 = 5 nM) and CDK9 (IC50 = 26 nM).^105^ Fadraciclib exhibited an approximately 34-fold anti-proliferative activity than that of seliciclib in a human tumor cell line panel.^105^ Fadraciclib induced a rapid and robust decrease in pSer2-RNAP II, loss of MCL-1, and decrease in RB pThr821 (a potential target of CDK2), and then led to cell death within a few hours.^105^ Percentage of cells in sub-G1 increases significantly 24 h after fadraciclib treatment in Colo205 cells.

Thus, CDK2/9 inhibitors are promising treatments for TNBC. Elevation of CDK2/cyclin E in a large proportion of TNBC and high expression of MYC contribute to the aggressive and refractory nature of TNBC tumors.^124^ Fadraciclib can inhibit CDK2 activity and deplete transcripts (such as MCL-1 and MYC) with a short half-life as a result of CDK9 inhibition. Fadraciclib induces apoptosis in TNBC cell lines (Cal51 and MDA-MB-468) and the HER2-amplified HCC1954 breast cancer cell line.^105^ Only solid tumor cell lines with high MCL-1 expression or a high MCL-1:BCL2L1 ratio, such as A2780 (ovarian cancer) and H23 (NSCLC), are sensitive to fadraciclib.^105^

Given that MCL-1 is essential for AML survival,^73,125^ most AML cell lines are highly sensitive to fadraciclib treatment. The proliferation of AML cells was inhibited by up to 90% after the cells were treated with low micromolar or sub-micromolar concentration of fadraciclib for 6 h.^105^ Sensitivity to fadraciclib was found to increase in AML and ALL cell lines carrying MLLr/MLL-PTD, which may be due to their dependence on the MLL-mediated transcription of HOXA9/MEIS1. Additionally, AML cell lines with MLLr/MLL-PTD lost HOXA9 after treatment with fadraciclib, which may also contribute to proliferation suppression.^105^ For tumors not dependent on the depletion of MCL-1, including some AML cell lines HEL, THP-1, and PL21, ABT-263/ABT-737, or venetoclax (BCL2 inhibitor) in combination with fadraciclib was effective at inducing apoptosis.^105^

In CLL, inhibition of CDK9 by fadraciclib reduced pSer2-RNAP II and blocked transcription in vitro, resulting in MCL-1 depletion and cell apoptosis. In addition, fadraciclib was synergistic with the BCL2 antagonist, venetoclax, inducing more profound CLL cell death, especially in samples with 17p deletion.^113^ CDK9 bound to the MYCN-amplicon super-enhancer as a component of P-TEFb; therefore, fadraciclib suppressed MYCN transcription, leading to growth arrest and apoptosis. Fadraciclib, in combination with temozolomide (used for therapy-resistant NB), caused long-term suppression of NB growth in vivo.^114^

Significantly, fadraciclib is currently undergoing a phase I clinical study in patients with advanced solid tumors, including endometrial or ovarian cancer, biliary tract cancer, HCC, breast cancer, and patients with relapsed or refractory CLL, AML, and myelodysplastic syndrome (MDS).

#### KB-0742

KB-0742, designed by Richters et al., is a selective ATP-competitive CDK9 inhibitor.^56^ It shows high selectivity against CDK9 with more than 50-fold selectivity over all CDKs profile and more than 100-fold selectivity against cell-cycle CDKs (CDK1–6) (IC50 = 6 nM at 10 μM ATP condition).^56^ KB-0742 (1.2 μM) was sufficient to reduce RNAP II Ser2 phosphorylation and induce growth arrest of 22rv1 PCa cells.

In contrast to NVP-2 and AZD4573, KB-0742 modulated cell apoptosis only at high doses (3 and 10 μM). CDK9 can prolong the half-life and activity of AR by phosphorylation at its N-terminal (Ser81),^126^ and KB-0742 can decrease the expression of AR through CDK9-mediated transcriptional regulation of the AR locus. Additionally, KB-0742 downregulates AR nascent transcription and disrupts AR-driven transcriptional programs and upstream master regulators (such as SOX4) in PCa.^56,127^ At an oral dose of 25–30 mg/kg daily, xenograft mouse models showed good tolerance and a significant reduction in tumor burden.^56^

#### LZT-106

The 4-carbonyl group of LZT-106 can form hydrogen bonds with Cys106 in the hinge region, enabling its embedding in the ATP-binding site of CDK9. LZT-106 exhibited 30-fold preferential kinase inhibitory activity against CDK9 compared to CDK2 (IC 50 = 30 nM vs. 1180 nM, respectively).

Exposure to LZT-106 reduces the expression of MCL-1 and facilitates degradation of MCL-1 via regulating GSK-3β signal pathway in CRC cells, leading to CRC cell apoptosis. Moreover, LZT-106 synergized with the BCL2 inhibitor ABT-199 to enhance ABT-199-induced apoptosis and death in RKO cells. Tumor growth in RKO xenograft mouse models was significantly inhibited by LZT-106 without obvious toxic effects.^55^

#### Vip152 (bay 1,251,152)

To improve the selectivity of atuveciclib to CDK9 under high concentrations of ATP and to avoid side effects, Lucking et al. modified the structure of atuveciclib and developed VIP152.^106^ They retained sulfoximine at the initial position to exhibit metabolic stability and high hydrolytic activity. They replaced triazine core of atuveciclib with 5-fluorine pyridine and substituted aromatics with pyridine to enable its intravenous administration.^106^ VIP152 binding to P-TEFb leads to its dissociation from the 7SK snRNP complex and inhibits P-TEFb binding to RNAP II, resulting in the loss of proliferative signaling and MCL-1 expression.^128^

VIP152 has the ability to inhibit growth and induce cell apoptosis in ovarian cancer, AML, and CLL and has been shown to have good efficacy and tolerance in human xenograft tumor models of AML and CLL.^106,128^ VIP152 has entered phase I clinical trials in patients (NCT02635672 and NCT02745743). VIP152 demonstrated a substantial safety profile with primary grade 1/2 adverse events and only one to two serious side effects occurring in one to two cases.^17^ The report on phase I clinical trial, NCT02635672, showed seven of 30 solid tumor patients got benefits from VIP152 and two of seven patients with high-grade B-cell lymphoma with MYC and BCL2/BCL6 translations achieved durable complete metabolic remission.^17^ Overall, VIP152 demonstrated promising therapy potency and safety profile in both patients with solid tumor and HGL.

### 4.2. CDK9 degraders

PROTACs have emerged as a new modality of chemical tools and potential therapeutic strategies to treat human diseases, especially cancers.^129,130^ PROTAC molecules consist of three components – a binding ligand for the protein of interest (POI), an E3 ubiquitin ligase-binding ligand, and a linker to connect two chemical scaffolds. Therefore, PROTACs are engineered to utilize the ubiquitin-proteasome system in living cells, which induces the degradation of POI. Since the kinase domains (especially the ATP-binding site) of CDKs are structurally similar, it is challenging to develop selective CDK inhibitors. However, the shape of the surface and the distribution of lysine residues on the surface among CDKs are different, which provides an opportunity to design a selective degrader based on the PROTAC strategy.^131^ Several CDK9 degraders, including THAL-SNS-032 and Wogonin-based PROTAC 11c, have been demonstrated to be efficient PROTAC degraders targeting CDK9, showing different pharmacological effects between inhibitors and degraders.

#### THAL-SNS-032

SNS-032, initially named BMS-387032, was reported as a selective inhibitor of CDK2/Cyclin E, which possesses antitumor activity in mice.^132^ It was later proven to be an inhibitor of CDK2, CDK7, and CDK9 and affected the transcription process.^133–135^ The main mechanism of SNS-032 action is to inhibit the phosphorylation of RNAP II and RNA synthesis.

THAL-SNS-032, a novel selective CDK9 degrader (PROTAC), was designed by derivatizing the solvent-exposed piperidine nitrogen of SNS-032 with a 3-polyethylene glycol (PEG) linker conjugated to thalidomide, which binds the E3 ubiquitin ligase cereblon (CRBN). Compared with SNS-032, although they have similar kinase-binding profiles, THAL-SNS-032 selectively induces degradation of CDK9, with only CDK9 exhibiting more than two-log-fold significant downregulation.^107^ THAL-SNS-032 displayed profound inhibitory activity in MCF7, T47D, and BT474 breast cancer cell lines. It does not affect the cell cycle, but mediates caspase3-induced apoptosis.^108^ However, because of the high expression of CDK9 in the gastrointestinal epithelium even at the lowest dose (2.5 mg/kg every 3 days), THAL-SNS-032 induced severe gastrointestinal disorders in mouse models due to an on-target off-tumor effect of the compound. Therefore, targeted delivery of THAL-SNS-032 to cancer cells is essential to open up the possibility of using this compound in treating cancer.^108^

#### 11c

11C is a selective CDK9 degrader designed based on the CDK9 inhibitor, wogonin. A linker with a triazole group conjugated wogonin to pomalidomide. It showed sub-micromolar biochemical inhibition of CDK9 (IC50 = 523 ± 12 nM). Compound 11c degraded CDK9 in a dose-dependent manner with unchanged CDK2, CDK4, CDK5, CDK7, and CDK8 levels, indicating selective CDK9 degradation. It inhibited the proliferation of CDK9-rich MCF7 cells and induced apoptosis in MCF7 cells in a dose-dependent manner by decreasing MCL-1 expression, whereas it was much less active against the CDK9 low-expressing cell line L02.^136^

#### B03

B03, another CDK9 degrader (DC50 = 7.62 nM) that utilizes the ubiquitin-proteasome system, can induce the degradation of CDK9 both in vitro and in vivo. Similar to THAL-SNS-032, B03 was designed based on the selective CDK9 inhibitor, BAY1143572, and conjugated BAY1143572 through a solvent-facing group via an alkyl triazole linker to recruit the CRL4^CRBN^ ubiquitin ligase complex. B03 degraded CDK9 and MCL-1 in a time-dependent manner and reduced the phosphorylation levels of Ser2 and Ser5 within the RNAP II CTD.^109^

Because of the slight increase in CDK9 inhibitory activity and degradation of CDK9, B03 exhibited significantly better anti-proliferative activity against MV4–11 than BAY1143572, indicating that its degradation activity is superior to its inhibitory effect.^109^ B03 exerts anti-proliferative activity against cancer cell lines (MV4–11, MOLM13, HCT-116, B16, A549, HepG2, and MCF7).^109^

#### Protac 2

PROTAC 2 is an aminopyrazole-based selective CDK9 degrader with a sub-micromolar DC50 value (158 ± 6 nM) that induces complete CDK9 degradation at 1 μM. PROTAC 2 forms a ternary complex with CDK9 and CRBN E3-ligase to induce ubiquitination-mediated proteasomal degradation of CDK9. Additionally, PROTAC2 potently sensitizes the pancreatic cancer cell line MiaPaCa2 to the BCL2 inhibitor, venetoclax.^110^ Resistance to BCL2 has been attributed to the compensatory activity of MCL-1; therefore, MCL-1 inactivation sensitizes cancer cells to BCL2 inhibitors.

#### Compound 45

Compound 45, which binds to both CDK9 and E3 ubiquitin ligases, showed high selectivity toward the degradation of CDK9 protein (IC50 = 4 nM). It did not induce the degradation of other CDK proteins, including CDK1, CDK2, CDK4, CDK5, CDK6, and CDK7, at concentrations ranging from 100 to 500 nM in MDA-MB-231 cells.^57^ Compound 45 could markedly reduce CDK9 and MYC, inhibiting the growth of TNBC cells (MDA-MB-468, MDA-MB-231, and BT-549) in vitro and tumor growth of MDA-MB-231 xenografts in vivo.^57^

Currently, there are far fewer reports on CDK9 degraders than on CDK9 inhibitors, including studies on mechanisms, in vitro and in vivo experiments as well as synergistic effects with other drugs. THAL-SNS-032, which has been relatively extensively studied among CDK9 degraders, has been reported to induce severe gastrointestinal disorders in mouse models owing to an on-target off-tumor effect. None of these CDK9 degraders has progressed to clinical trials, and further research is needed on PROTACs as CDK9 degraders.

## 5. Discussion

CDK9 promotes transcriptional elongation through the release of RNAP II pause. Additionally, CDK9 is essential for the maintenance of heterochromatin compaction.^8^ Since it was discovered more than 20 years ago, CDK9 has been shown to be a key player in several diseases as well as a variety of cancers and targeting CDK9 is considered as a promising therapeutic strategy. While continuing efforts to optimize the specificity of CDK9 inhibitors, the stability, cytotoxicity, side effects, and tolerability of the inhibitors have also been considered. In recent times, more selective and potent CDK9 inhibitors and degraders have emerged.

Most selective CDK9 inhibitors, such as AZD4573, atuveciclib, VIP152, A-1592668 and JSH-150, have been shown to reduce tumor growth, mainly in hematological malignancies, in vitro and in vivo, and some of them have already progressed to phase I clinical trials (Table 1). Recently, increased attention has been paid to the study of CDK9 inhibitors in solid tumors. NVP-2, MC180295, fadraciclib, KB-0742, LZT-106, and 21e have been developed, and the inhibitory effects of NVP-2 on BRAF^wt^/NRAS^wt^/NF1^wt^ cutaneous and uveal melanomas, MC180295 on CRC, fadraciclib on MYCN-amplified Kelly NB tumor, KB-0742 on Pca, LZT-106 on CRC, and 21e on NSCLC, have been reported. Among them, KB-0742 has progressed to phase I clinical trial for relapsed or refractory solid tumors or non-Hodgkin’s lymphoma (Table 1).

**Figure 6.**
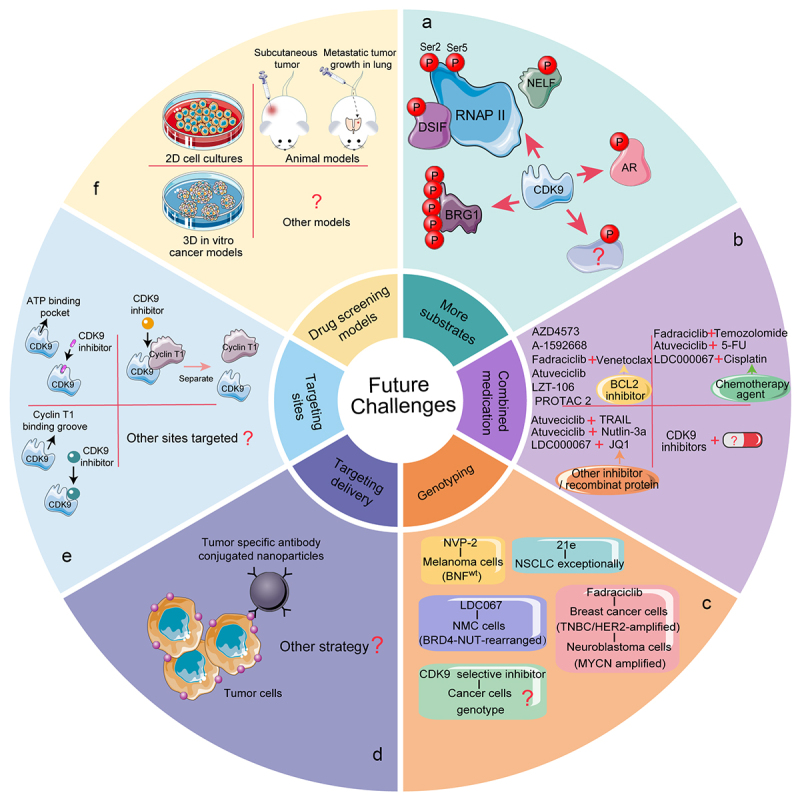
Future prospects for CDK9 and its selective inhibitors and degraders.

Although these reports are encouraging in that selective CDK9 inhibitors and degraders have been demonstrated to restrain tumor growth in hematological malignancies and solid tumors in vitro and in vivo, many problems still remain. 1) To date, only a few phosphorylation substrates for CDK9 have been reported, including Ser2-RNAP II, the C-terminal domain of BRG1, and AR Ser81. With the rapid development of protein phosphorylation research technologies, such as 4D label-free phosphorylation quantitative proteomics, we believe that more phosphorylation substrates for CDK9 will be explored in the future. Currently, known mechanisms, such as MCL-1 dysregulation triggered by CDK9 in hematological malignancies and solid tumors, have mainly been discovered using pan-CDK inhibitors. With the development of selective CDK9 inhibitors and degraders, we anticipate that additional mechanisms involved in CDK9 dysregulation, such as BRG1, will be found (Figure 6a). 2) Synergistic effects of CDK9 inhibitors with venetoclax, temozolomide, TRAIL, Nutlin-3a, cisplatin, and JQ1 on leukemia and solid tumor cells have been explored, largely with venetoclax, and promising results have been reported (Table 2). The synergistic effects of a CDK9 inhibitor with a BCL2 inhibitor can prevent the compensatory increase in MCL-1, whose expression increases due to BCL2 blockade. Recently, it was reported that combining PROTAC with a drug efflux protein (MDR) inhibitor can achieve durable protein degradation and a therapeutic response in cancer.^137^ Combination therapy may be a promising strategy to improve the efficacy of selective CDK9 inhibitors and degraders in tumors. Additional combination regimens of CDK9 inhibitors and other drugs should be explored in the future (Figure 6b). 3) Some effective inhibitors of solid tumors require specific genes or receptors to exhibit efficacy. For example, fadraciclib only works on solid tumor cell lines with high expression of MCL-1 or a high MCL-1: BCL2L1 ratio. KB-0742 downregulates AR expression, resulting in a promising therapeutic strategy for Pca, fadraciclib suppresses MYCN transcription, leading to growth arrest and apoptosis of MYCN-amplified NB. Tumor heterogeneity is one of the most important characteristics of malignant tumors, including tumor and intratumoral heterogeneity, which suggests that the genes and phenotypes of cells in different tumors, and in different cells of the same tumor, are different, especially in solid tumors. Further studies of CDK9 inhibitors in tumors should rely on genotyping (Figure 6c). 4) It has been reported that even at low-doses, CDK9 degrader THAL-SNS-032 induced severe gastrointestinal disorders in mouse models due to an on-target off-tumor effect of the compound because of the high expression of CDK9 in the gastrointestinal epithelium. Therefore, the specific delivery of CDK9 degraders to cancer cells could open up the possibility of using this compound in cancer treatment. One solution is to formulate inhibitors or degraders in nanoparticles to enable targeted delivery of CDK9 inhibitors or degraders to solid tumors. Noblejas-López et al. demonstrated the use of bromo and extraterminal domain (BET) inhibitors-PROteolysis Targeting Chimera (BETi-PROTAC) inhibitor – MZ1—in a nanoparticle-conjugated trastuzumab to treat breast cancer (Figure 6d).^138^ Identifying proteins or receptors that are only expressed in specific tumor types and not in normal tissues will assist in improving delivery efficacy and avoiding side effects. 5) The high structural homology of the ATP-binding pocket among CDKs makes the development of highly selective CDK9 kinase inhibitors challenging. Currently, the most well-known CDK9 inhibitors are nonselective inhibitors. Since the kinase domains and ATP-binding sites of CDKs are structurally similar, non-ATP competitive mechanisms should be considered, such as blocking substrate recruitment and interfering with CDK-cyclin interactions (Figure 6e).^139^ 6) The development of new drugs requires suitable drug screening models, which commonly include 2D cell cultures, animal models, and 3D cancer models (Figure 6f). Compared with 2D cell cultures, animal models can reflect the spatial structure of in vivo tissues and the situation in the tumor microenvironment, which makes the results more reliable.^140,141^ However, the animal tumor microenvironment still cannot completely simulate the human tumor microenvironment, and animal experiments are subject to ethical limitations; therefore, 3D cancer models are being developed. In vitro 3D cancer models can improve the predictability of toxicity and drug sensitivity in cancer.^142^ Unfortunately, 3D cancer models still do not meet the standards required for preclinical trials. Therefore, it is challenging to find drug screening models that can effectively simulate the human tumor microenvironment and meet the criteria for preclinical trials. 7) The past two decades have witnessed progress in targeted therapies closely tied to technological advancements in sequencings, such as next-generation sequencing (NGS). The exploitation of genetic mutations in CDK9 will benefit CDK9 targeted therapies and limit the potential for off-target toxicity.

A. Five phosphorylation substrates of CDK9 have been demonstrated, and more substrates await development. B. Three kinds of agents and a total of seven agents are reported to synergize with CDK9 inhibitors or degraders in antitumor therapy; more synergistic drugs are required to be identified. C. Four selective CDK9 inhibitors are confirmed to act on tumors with specific genotypes, so tumor genotypes targeted by CDK9 inhibitors require more attention. D. Tumor specific antibody conjugated nanoparticles may help targeting delivery of CDK9 inhibitors to tumor cells, and other targeted delivery strategies need to be further developed. E. ATP non-competitive mechanisms involved in development of CDK9 inhibitors should attract more attention, such as blocking the substrate recruitment and interfering with CDK-cyclin interaction. F. More drug screening models other than 2D cell cultures, animal models and in vitro 3D cancer models should be developed.

In conclusion, most CDK9 inhibitors and degraders have only been studied in cancer cells in vitro or in mouse models in vivo, and few clinical studies have been completed owing to drug side effects. As a potential tumor target, there are still many problems to be solved before CDK9 inhibitors and degraders can be applied clinically.

## Data Availability

Not applicable.

## Abbreviations

CDK: cyclin-dependent kinase
CDK9: cyclin-dependent kinase 9
RNAP II: RNA polymerase II
P-TEFb: positive transcription elongation factor b
CTD: C-terminal domain
ER: estrogen receptor
HER2: human epidermal growth factor receptor 2
DSIF: DRB sensitivity inducing factor
NELF: negative elongation factor
HEXIM: hexamethylene bisacetamide-inducible proteins
BRG1: Brahma-related gene 1
mRNA: messenger RNA
HCC: hepatocellular carcinoma
PCa: Prostate cancer
PDX: patient-derived xenograft
DLBCL: diffuse large B-cell lymphoma
FL: follicular lymphoma
CLL: chronic lymphocytic leukemia
AML: acute myeloid leukemia
MDS: myelodysplastic syndrome
T-PLL: T-cell prolymphocytic leukemia
PDAC: pancreatic ductal adenocarcinoma
MB: medulloblastoma
CRC: colorectal cancer
NSCLC: non-small cell lung cancer
HGL: high-grade B-cell lymphoma with MYC and BCL2/BCL6 translocation
TNBC: triple-negative breast cancer
NMC: nut midline carcinoma
MM: multiple myeloma
AR: androgen receptor
PROTAC: proteolysis-targeting chimera
NB: neuroblastoma
PEL: primary effusion lymphoma
GISTs: gastrointestinal stromal tumors
POI: protein of interest
CRBN: cereblon
MLL: mixed lineage leukemia
MCL-1: myeloid cell leukemia 1
TCGA: the Cancer Genome Atlas
KSHV: Kaposi’s sarcoma-associated herpesvirus
BET: bromo and extraterminal domain
EAC: esophageal adenocarcinoma
CTSK: cathepsin K
SCLC: small cell lung cancer
NGS: next-generation sequencing
